# Rashba spin splitting and phonon-limited carrier mobility in novel two-dimensional Janus ZCrBSe_2_ (Z = N, P, As) materials

**DOI:** 10.1039/d6ra00580b

**Published:** 2026-03-19

**Authors:** Tuan V. Vu, A. I. Kartamyshev, Bui D. Hoi, Huynh V. Phuc, Cuong Q. Nguyen

**Affiliations:** a Laboratory for Computational Physics, Institute for Computational Science and Artificial Intelligence, Van Lang University Ho Chi Minh City Vietnam tuan.vu@vlu.edu.vn; b Faculty of Mechanical, Electrical, and Computer Engineering, Van Lang School of Technology, Van Lang University Ho Chi Minh City Vietnam; c Faculty of Physics, University of Education, Hue University Hue Vietnam; d Division of Physics, School of Education, Dong Thap University Dong Thap 870000 Vietnam; e Institute of Research and Development, Duy Tan University Da Nang 550000 Vietnam nguyenquangcuong3@duytan.edu.vn; f School of Engineering & Technology, Duy Tan University Da Nang 550000 Vietnam

## Abstract

In this study, we employ density functional theory to systematically investigate the properties of novel Janus ZCrBSe_2_ (Z = N, P, As) monolayers. These two-dimensional semiconductors are found to be dynamically and thermally stable and to possess narrow band gaps. Owing to their intrinsic structural asymmetry and the resulting breaking of out-of-plane inversion symmetry, spin–orbit coupling not only induces Zeeman-type spin splitting but also gives rise to pronounced Rashba-type spin splitting in all proposed Janus structures. Furthermore, we comprehensively analyze the role of different phonon scattering mechanisms in determining the carrier mobility. The results demonstrate that acoustic deformation potential scattering is the dominant limiting factor and plays a decisive role in setting the total carrier mobility over a wide range of conditions. Beyond intrinsic phonon scattering, carrier concentration is shown to have a significant impact on mobility behavior, particularly for ionized impurity scattering, whose associated mobility exhibits a strong dependence on carrier density. These findings highlight the intricate interplay between intrinsic lattice vibrations and extrinsic impurity effects in governing carrier transport in Janus two-dimensional materials.

## Abstract 2

In this study, we employ density functional theory to systematically investigate the properties of novel Janus ZCrBSe_2_ (Z = N, P, As) monolayers.

## Graphical abstract

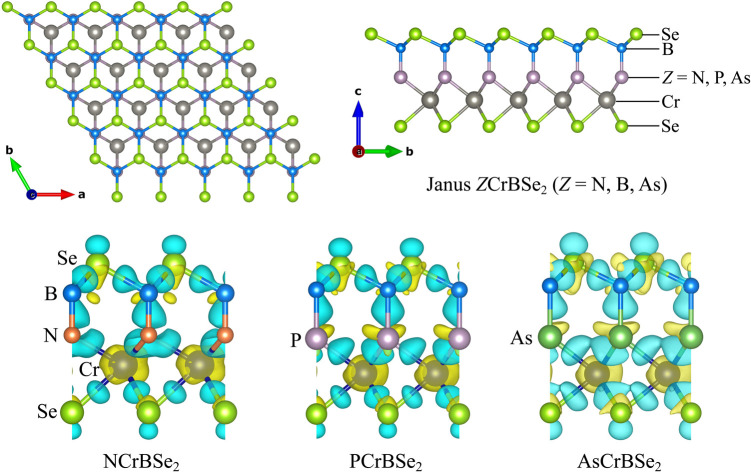

## 1 Introduction

Two-dimensional (2D) materials have garnered significant interest due to their exceptional electronic, optical, and mechanical properties resulting from their reduced dimensionality.^1,2^ These characteristics position them as prime candidates for next-generation nanoelectronic and optoelectronic applications.^3^ Among them, Janus 2D materials, characterized by intrinsic structural and chemical asymmetry across the out-of-plane direction, provide an additional degree of freedom for engineering material properties, such as built-in electric fields, strengthened spin–orbit coupling, and anisotropic carrier transport behavior.^4–6^ In particular, multicomponent Janus materials incorporating pnictogen elements have attracted growing interest, as pnictogens enable versatile bonding configurations, highly tunable band structures, and pronounced spin–orbit interactions, which are essential for achieving high-performance spintronic functionalities.^7–9^ A vital metric for device performance is carrier mobility, which dictates charge transport efficiency and switching speeds. It was demonstrated that mobility in these materials is primarily limited by phonon scattering at room temperature. The interaction between charge carriers and acoustic/optical phonons dissipates momentum and energy, defining the fundamental limit of mobility.^10^ Consequently, a systematic study of phonon-limited carrier mobility in pnictogen-based multicomponent Janus 2D materials is crucial for evaluating their potential in practical electronic devices.^11,12^

Owing to their reduced dimensionality, 2D materials exhibit weakened short-channel effects and enhanced electrostatic control, making carrier mobility a decisive factor in pushing device performance beyond the scaling limits of conventional silicon-based electronics. From a theoretical perspective, carrier mobility is commonly estimated using the deformation potential (DP) theory originally developed by Bardeen and Shockley.^13^ Owing to its relatively low computational cost, this approach has been extensively applied to 2D materials, as it models carrier scattering by considering only long-wavelength acoustic phonons and approximates electron–phonon interactions as rigid shifts of the electronic bands under lattice deformation. Despite its widespread use, the DP framework suffers from notable limitations. In particular, it tends to overestimate carrier mobility because it disregards several important scattering channels, including polar optical phonon (POP) scattering, ionized impurity scattering, and piezoelectric scattering. For many 2D semiconductors, especially at room temperature, these omitted mechanisms, most notably POP scattering, play a dominant role in constraining charge transport.^10,14^ In 2D materials, phonon-induced scattering constitutes the dominant intrinsic factor restricting carrier mobility at room temperature.^15^ During charge transport, interactions between charge carriers and lattice vibrations lead to frequent scattering events, resulting in momentum relaxation and energy dissipation. A precise and comprehensive description of these phonon-related scattering processes is therefore crucial for reliably predicting carrier transport behavior and evaluating the performance of 2D semiconductors in practical electronic applications.^15^

To overcome the inherent limitations of the deformation potential approximation, the AMSET has been developed as a more rigorous and comprehensive theoretical framework.^16^ In contrast to the simplified DP approach, AMSET explicitly incorporates multiple carrier scattering mechanisms based on quantities obtained directly from first-principles electronic-structure calculations. These mechanisms typically include acoustic deformation potential scattering, polar optical phonon scattering, ionized impurity scattering, and piezoelectric scattering, allowing for a more realistic description of charge transport. By evaluating energy- and momentum-dependent scattering rates without relying on empirical fitting parameters, AMSET provides a quantitatively reliable estimation of carrier mobility and thermoelectric transport coefficients over a wide range of temperatures and carrier concentrations. As a result, this method offers a significant improvement in predictive accuracy, particularly for polar and low-dimensional materials where electron–phonon interactions beyond acoustic phonons play a decisive role.^17,18^

In this study, we systematically investigate the impact of various phonon scattering mechanisms on the carrier mobility of novel Janus ZCrBSe_2_ (Z = N, P, As) monolayers using the AMSET software package. In addition to transport properties, first-principles density functional theory (DFT) calculations are employed to provide a consistent description of the crystal structures and electronic band characteristics. Particular emphasis is placed on elucidating the role of spin–orbit coupling (SOC), which is explicitly accounted for to evaluate its influence on band dispersion, band-gap renormalization, and the resulting charge transport behavior. This integrated approach facilitates a deeper understanding of the intrinsic mechanisms governing transport in Janus ZCrBSe_2_ monolayers and offers valuable insights into their potential for electronic and spintronic applications.

## 2 Computational details

All first-principles calculations were carried out using DFT method as implemented in the Vienna *ab initio* Simulation Package (VASP).^19,20^ Electron–ion interactions were treated *via* the projector augmented wave (PAW) method.^21,22^ For the plane-wave expansion, a kinetic energy cutoff of 650 eV was employed to ensure high precision, while the Brillouin zone was sampled using a 12 × 12 × 1 Monkhorst–Pack *k*-mesh. To simulate an isolated monolayer and prevent spurious interactions between periodic images, a generous vacuum layer of 30 Å was added along the *z*-axis. Structural relaxation was carried out until the interatomic forces subsided below 10^−3^ eV Å^−1^, with a tight energy convergence threshold of 10^−6^ eV for the electronic steps. Furthermore, dipole corrections were included to properly capture the built-in electric field induced by the absence of mirror symmetry in the Janus materials. Additionally, dipole corrections were included to properly capture the built-in electric field induced. The dynamical stability of the systems was verified by computing phonon spectra with the finite-displacement method in PHONOPY,^23^ utilizing a 5 × 5 × 1 supercell expansion for convergence. Complementary to this, the structural integrity at finite temperatures was explored using *ab inito* molecular dynamics (AIMD) within the NVT ensemble. Electronic structures were modeled using a dual-functional approach: the generalized gradient approximation (GGA-PBE)^24^ was used for general trends, while the Heyd–Scuseria–Ernzerhof (HSE06) hybrid functional^25^ was applied to mitigate the self-interaction error and improve band-structure accuracy. To account for relativistic effects essential for these systems, SOC was explicitly considered in the electronic Hamiltonian.^26^ It is also noted that although introducing a Hubbard U term can improve the quantitative description of magnetic moments, the primary objective of this work is to identify systematic structural and electronic trends across the pnictogen series. Therefore, the Hubbard correction was not included in our calculations. The transport properties were scrutinized using the AMSET code,^16^ which leverages ground-state electronic structure data from VASP to compute transport coefficients. Multiple carrier scattering channels were considered, including acoustic deformation potential (ADP), ionized impurity (IMP), piezoelectric (PIE), and polar optical phonon (POP) scattering. To derive the total mobility from these decoupled mechanisms, we applied Matthiessen's rule,^10^ which assumes that the total resistivity is the sum of the resistivities from each independent scattering process.

## 3 Results and discussion

### 3.1 Crystal structure and stability

**Fig. 1:**
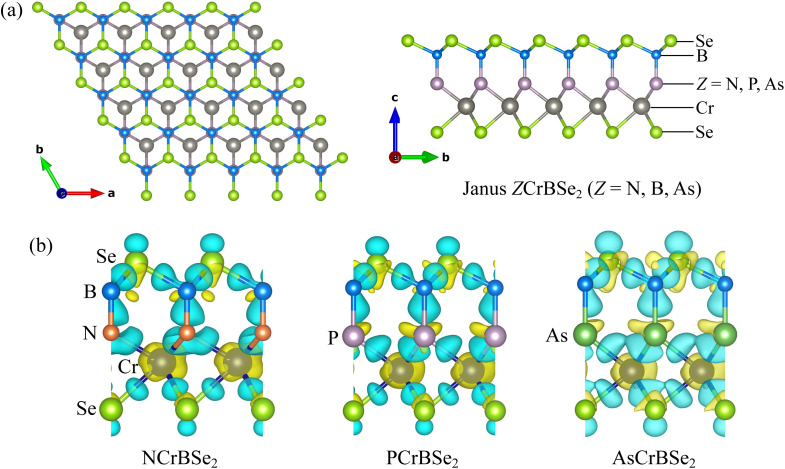
(a) Different views of the crystal structure and (b) charge density difference of 2D Janus ZCrBSe_2_ (Z = N, P, As) structures.

The ground-state crystal structures of the Janus ZCrBSe_2_ (Z = N, P, As) monolayers are illustrated in Fig. 1(a). This family of 2D materials adopts a hexagonal lattice characterized by a distinct structural asymmetry along the out-of-plane direction. Each monolayer is composed of five atomic sublayers arranged in a Se–Cr–Z–B–Se sequence. In this configuration, the outer Selenium layers sandwich the inner Cr, and Z, and B layers. The vertical inequivalence of the atomic species inherently breaks both mirror and inversion symmetries, leading to a non-centrosymmetric stacking sequence. As a result, these monolayers belong to the polar space group *P*3*m*1. This specific symmetry class is of particular interest as it often facilitates unconventional electronic responses, significant piezoelectricity, and pronounced SOC effect.

Following first-principles structural relaxation, the optimized in-plane lattice constants (*a*) were determined to be 3.08, 3.20, and 3.24 Å, respectively. A clear monotonic expansion of the lattice is observed as the pnictogen species progresses from N to As, a trend directly attributable to the increasing atomic radii of the pnictogen elements. Comprehensive structural data, including interatomic bond lengths (*d*) and monolayer thickness (Δ*h*), are detailed in Table 1. As shown in Table 1, the Se–B and Cr–Se bond lengths exhibit only negligible variations among the three Janus structures of ZCrBNSe_2_, indicating that the local bonding environment associated with Se atoms remains relatively insensitive to the choice of the pnictogen element. In contrast, the B–Z and Cr–Z bond lengths display a clear increasing trend as the atomic radius of Z increases from N to P and As. This behavior can be directly attributed to the progressively larger size of the pnictogen atoms, which leads to an expansion of the corresponding bonds.

**Table 1:** Lattice constant *a* (Å), chemical bond length *d* (Å), monolayer thickness Δ*h* (Å), cohesive energy *E*_coh_ (eV per atom), elastic constants *C*_*ij*_ (N m^−1^), Young's modulus *Y*_2D_ (N m^−1^), and Poisson's ratio *ν*_2D_ of 2D Janus ZCrBSe_2_ (Z = N, P, As) materials

	*a*	*d*_Se–B_	*d*_B–Z_	*d*_Z–Cr_	*d*_Cr–Se_	Δ*h*	*E*_coh_	*C*_11_	*C*_12_	*C*_66_	*Y*_2D_	*ν*_2D_
NCrBSe_2_	3.08	2.04	1.45	2.06	2.42	5.15	−6.52	224.15	50.63	86.76	212.71	0.23
PCrBSe_2_	3.20	2.07	1.88	2.28	2.43	5.75	−5.99	183.32	46.40	68.46	171.58	0.25
AsCrBSe_2_	3.24	2.09	2.03	2.38	2.43	5.97	−5.72	173.93	45.09	64.42	162.25	0.26

To further elucidate the chemical bonding characteristics and charge redistribution in these systems, the charge density differences of the NCrBSe_2_, PCrBSe_2_, and AsCrBSe_2_ monolayers are calculated and presented in Fig. 1(b). The plots clearly reveal the redistribution of electronic charge induced by bond formation. For all three monolayers, electron density is mainly concentrated near the Se and B sites, whereas notable charge depletion is observed in the vicinity of the Cr and Z (N, P, As) atoms. This behavior indicates the presence of polar covalent bonding, characterized by substantial charge transfer from the electropositive Cr and pnictogen atoms toward the more electronegative Se and B atoms. As the pnictogen species evolves from N to P and As, the charge redistribution near the Z sites becomes increasingly diffuse, which can be attributed to the larger atomic radius and lower electronegativity of the heavier pnictogens. As a result, the Cr–Z and B–Z bonds in AsCrBSe_2_ show reduced charge localization compared to those in NCrBSe_2_, implying a gradual weakening of bond ionicity and enhanced electronic delocalization. This systematic evolution of the charge density difference is consistent with the observed lattice expansion and underscores the crucial role of the pnictogen element in modulating the bonding nature of Janus CrBZSe_2_ monolayers.

We further evaluate the structural stability and bonding strength of the proposed systems by calculating the cohesive energy. The cohesive energy represents the energy required to separate a solid into its constituent isolated atoms and thus provides a quantitative measure of the strength of interatomic interactions within the material. It is typically determined from the difference between the total energy of the crystalline system and the summed energies of the corresponding free atoms, normalized per atom. A more negative cohesive energy signifies stronger chemical bonding and higher intrinsic structural stability. Accordingly, the calculation of cohesive energy provides a direct and reliable criterion for evaluating the energetic stability and feasibility of newly designed or theoretically predicted materials. Moreover, comparative analysis of cohesive energies among related compounds yields valuable insights into the effects of chemical composition and local bonding environments on structural robustness, which is critical for evaluating their potential for experimental realization and practical applications. The cohesive energy per atom *E*_coh_ of the Janus ZCrBSe_2_ can be calculated as the following:1
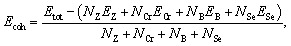
where *E*_tot_ indicates the total energy of the studied Janus ZCrBZSe_2_ sheet. The quantities *E*_Z_, *E*_Cr_, *E*_B_, and *E*_Se_ correspond to the energies of isolated Z, Cr, B, and Se atoms, respectively. The symbols *N*_Z_, *N*_Cr_, *N*_B_, and *N*_Se_ indicate the number of Z, Cr, B, and Se atoms contained in the calculated unit cell.

The cohesive energies of NCrBSe_2_, PCrBSe_2_, and AsCrBSe_2_ are calculated to be −6.52, −5.99, and −5.72 eV per atom, respectively, confirming that all three proposed Janus monolayers are thermodynamically stable relative to their isolated atomic constituents. Among them, NCrBSe_2_ possesses the most negative cohesive energy, indicating the strongest interatomic interactions and the highest structural stability. With the substitution of the pnictogen atom from N to P and further to As, the cohesive energy becomes progressively less negative, reflecting a gradual weakening of the chemical bonds. This behavior can be rationalized by the increasing atomic size and reduced electronegativity of the heavier pnictogen elements, which result in elongated Cr–Z and B–Z bonds and diminished orbital hybridization. As a consequence, AsCrBSe_2_ exhibits comparatively weaker bonding and lower structural robustness, although its cohesive energy remains sufficiently negative to guarantee overall stability. The observed monotonic decrease in cohesive energy underscores the decisive role of the pnictogen species in modulating bond strength and structural stability in Janus ZCrBSe_2_ monolayers, offering an effective strategy for stability engineering *via* compositional control.

**Fig. 2:**
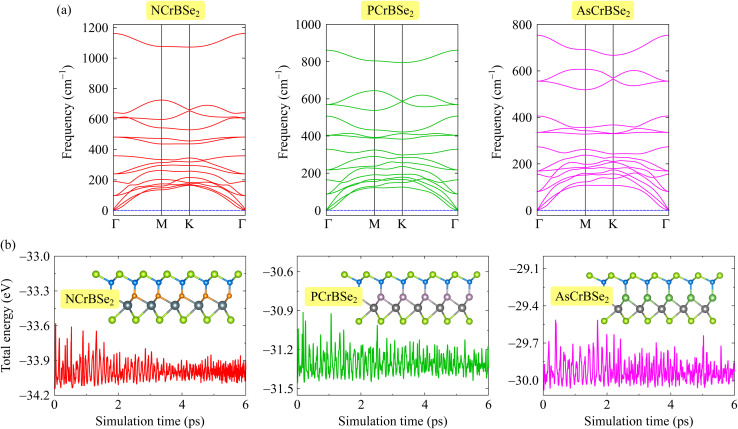
Calculated phonon spectrum (a) and AIMD simulations at room-temperature (b) of 2D Janus ZCrBNSe_2_ monolayers. Insets in (b) are the crystal structures of ZCrBSe_2_ monolayers at the end of the AIMD test.

We next calculate the phonon spectrum of the studied structures to investigate their dynamical stablity. The calculation of the phonon spectrum is essential for evaluating the dynamical stability of a material, as it directly reflects the lattice vibrational response to small atomic displacements. In general, a crystal is considered dynamically stable when its phonon dispersion exhibits no imaginary (negative) frequencies throughout the entire Brillouin zone. The absence of such modes indicates that the lattice can sustain small atomic displacements without undergoing spontaneous structural rearrangements. In contrast, the presence of imaginary phonon modes signifies dynamical instabilities, as these modes correspond to directions in configuration space along which the total energy of the system can be lowered. The occurrence of imaginary phonon frequencies indicates that the restoring forces counteracting atomic displacements are effectively suppressed. As a result, imaginary frequencies often imply a tendency toward structural distortions, lattice reconstructions, or even phase transitions into a more stable configuration. Therefore, phonon calculations provide a crucial criterion, complementary to energetic stability, for assessing whether a theoretically proposed structure can exist under realistic conditions. The calculated phonon spectra of the proposed Janus ZCrBSe_2_ monolayers are presented in Fig. 2(a). It is demonstrated that the obtained phonon dispersions show no imaginary frequencies across the Brillouin zone, confirming their dynamical stability. Fig. 2(a) further reveals a relatively broad frequency region in which acoustic and optical phonon modes overlap. Such coexistence facilitates strong acoustic–optical phonon scattering, enhancing phonon–phonon interactions and shortening phonon lifetimes. As a result, heat-carrying phonons are more effectively scattered, leading to a reduction in the lattice thermal conductivity of these materials. In the high-frequency optical region, clear phonon gaps emerge, which originate from the mass and bonding differences among the constituent atoms. These phonon gaps are indicative of vibrational mode separation and may play an important role in suppressing phonon–phonon scattering, thereby influencing thermal transport properties. Overall, the absence of imaginary modes, together with the presence of acoustic–optical mode hybridization and distinct phonon gaps, demonstrates the robust dynamical stability of the Janus ZCrBSe_2_ monolayers.

In addition to cohesive energy and phonon spectrum calculations, *ab initio* molecular dynamics (AIMD) simulations are necessary to provide a more comprehensive assessment of the structural stability of crystalline materials. While cohesive energy evaluates the strength of chemical bonds and phonon spectra probe dynamical stability at 0 K, these approaches do not fully capture the effects of finite temperature and anharmonic lattice vibrations. AIMD simulations explicitly account for thermal fluctuations and atomic motions at elevated temperatures, allowing one to examine whether the crystal structure can maintain its integrity over time under realistic thermal conditions. Therefore, AIMD serves as a crucial complementary tool to verify the thermal stability of the material and to confirm its feasibility for practical applications. The AIMD simulation results at room temperature for the ZCrBSe_2_ monolayers reveal that the total energy fluctuates only slightly, within about 0.5 eV, during the entire simulation time, as shown in Fig. 2(b). Such limited energy fluctuations indicate good thermal stability of the system. Furthermore, the final atomic configuration obtained at the end of the simulation retains the original crystal structure, exhibiting only negligible distortions and no signs of bond breaking or phase transition, as illustrated in insets of Fig. 2(b). These results confirm the structural robustness of the proposed Janus ZCrBSe_2_ monolayers under ambient conditions. Under realistic conditions, 2D materials are typically supported on substrates that can significantly influence their electronic properties *via* strain, dielectric screening, and interfacial hybridization. Such interactions often modify band gaps and spin-dependent features near the Fermi level. To preserve the monolayer's intrinsic characteristics, weakly interacting van der Waals substrates are preferred.

Further, we assess the mechanical stability of the materials through a detailed analysis of the calculated elastic constants. These constants are fundamental parameters that not only determine mechanical stability but also play a crucial role in describing and understanding other key mechanical properties of the materials. For hexagonal 2D systems, only two independent elastic constants, namely *C*_11_ and *C*_12_, are required to fully describe their elastic response. The *C*_66_ elastic constant is determined from *C*_11_ and *C*_12_ according to *C*_66_ = (*C*_11_ − *C*_12_)/2. The elastic constants of Janus ZCrBSe_2_ structures are summarized in Table 1. It is indicated that all elastic constants are positive and *C*_11_ − *C*_12_ > 0, which fully satisfies the Born mechanical stability criteria.^27,28^ This result clearly confirms the intrinsic mechanical stability of the proposed materials and demonstrates their ability to maintain structural integrity under small elastic deformations. Further insight into their mechanical robustness is obtained from the in-plane Young's modulus *Y*_2D_ and Poisson's ration *ν*_2D_. Among the studied systems, NCrBSe_2_ exhibits the highest Young's modulus, indicating the strongest in-plane stiffness and superior resistance to elastic deformation. In contrast, PCrBSe_2_ and AsCrBSe_2_ show moderately reduced *Y*_2D_ values, suggesting a gradual softening of the lattice with increasing atomic size of the Z element. The calculated Poisson's ratios for all three monolayers fall within a narrow range (from 0.23 to 0.26), implying typical elastic behavior and good structural flexibility without brittleness. Overall, the combination of high Young's modulus and moderate Poisson's ratio demonstrates that these Janus ZCrBSe_2_ monolayers possess good mechanical strength and elastic resilience, making them promising candidates for flexible two-dimensional electronic and nanoelectromechanical applications.

### 3.2 Band structures and Rashba-type spin-splitting

**Fig. 3:**
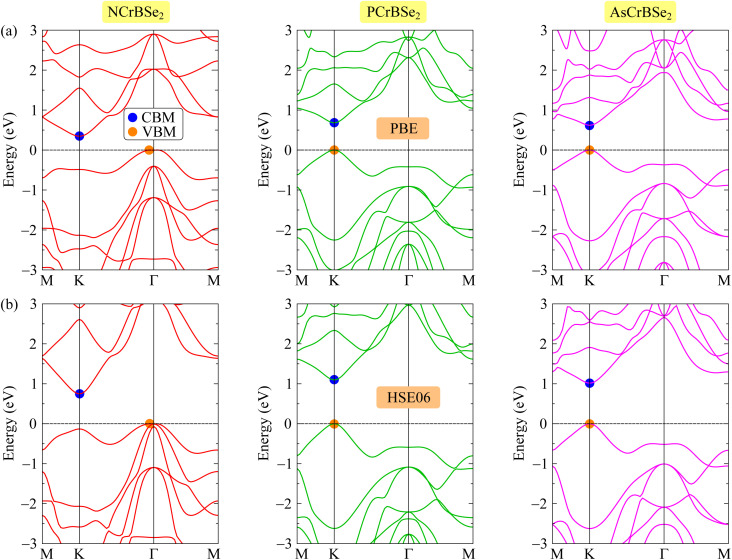
Band structures of 2D Janus ZCrBNSe_2_ (Z = N, P, As) materials at the PBE (a) and HSE06 (b) theoretical levels.

Here, we systematically present the calculated electronic band structures and provide a detailed analysis of the associated electronic characteristics, aiming to elucidate the underlying electronic behavior and its implications for the physical properties of the investigated systems. The electronic band structures of Janus ZCrBSe_2_ monolayers are shown in Fig. 3. The calculated results clearly indicate that all three materials exhibit semiconducting behavior. Specifically, NCrBSe_2_ is identified as an indirect-band-gap semiconductor, in which the valence band maximum (VBM) and the conduction band minimum (CBM) are located at the *K* point and the *Γ* point of the Brillouin zone, respectively. In contrast, both PCrBSe_2_ and AsCrBSe_2_ possess direct band gaps, with their VBM and CBM occurring at the same high-symmetry *K* point. The electronic band structures calculated using the PBE functional, as shown in Fig. 3(a), reveal that the NCrBSe_2_, PCrBSe_2_, and AsCrBSe_2_ monolayers are semiconductors with small band gaps of 0.35, 0.68, and 0.62 eV, respectively, as summarized in Table 2. In DFT calculations, the predicted band gap energy strongly depends on the choice of the exchange–correlation functional. The PBE functional is widely employed due to its computational efficiency and relatively low computational cost. However, it is well known to underestimate band gap values. To overcome this limitation, more advanced functionals have been developed, among which the HSE06 functional is commonly used. The calculated HSE06 band structures of ZCrBSe_2_ monolayers are illustrated in Fig. 3(b). It is demonstrated that the electronic band structures calculated using HSE06 functional exhibit a similar overall dispersion and qualitative features to those obtained with PBE, indicating that the band topology is preserved. Nevertheless, a significant difference is observed in the magnitude of the band gaps, which are substantially enlarged when using HSE06. Specifically, the HSE06 band gaps of NCrBSe_2_, PCrBSe_2_, and AsCrBSe_2_ monolayers are found to be 0.75, 1.10, and 1.02 eV, respectively, demonstrating the strong effect of the exchange–correlation treatment on band gap prediction.

**Table 2:** Calculated band gaps (*E*_g_) using the PBE, HSE06, and PBE+SOC methods; Zeeman splitting energy at *K* point in the valence band *λ*^v^_Z_(*K*); Rashba-type spin-splitting related parameters (*E*_R_, *k*_R_, and *α*_R_); vacuum level offset Δ*Φ*; and work functions (*Φ*_1,2_) of the proposed 2D Janus ZCrBSe_2_ materials

	*E* ^PBE^_g_ (eV)	*E* ^HSE06^_g_ (eV)	*E* ^PBE+SOC^_g_ (eV)	*λ* ^v^_Z_(*K*) (meV)	*E*_R_ *K* (meV)	*E*_R_^*M*^ (meV)	*k*_R_^*K*^ (Å^−1^)	*k*_R_^*M*^ (Å^−1^)	*α*_R_^*K*^ (meV Å)	*α*_R_^*K*^ (meV Å)	Δ*Φ* (eV)	*Φ*_1_ (eV)	*Φ*_2_ (eV)
NCrBSe_2_	0.35	0.75	0.33	79.4	10.0	9.4	0.083	0.072	240.96	261.11	0.07	4.52	4.35
PCrBSe_2_	0.68	1.10	0.64	81.2	9.0	8.8	0.185	0.172	97.30	102.33	1.47	4.85	3.38
AsCrBSe_2_	0.62	1.02	0.57	90.9	32.3	30.2	0.222	0.204	290.99	296.08	1.84	4.94	3.10

**Fig. 4:**
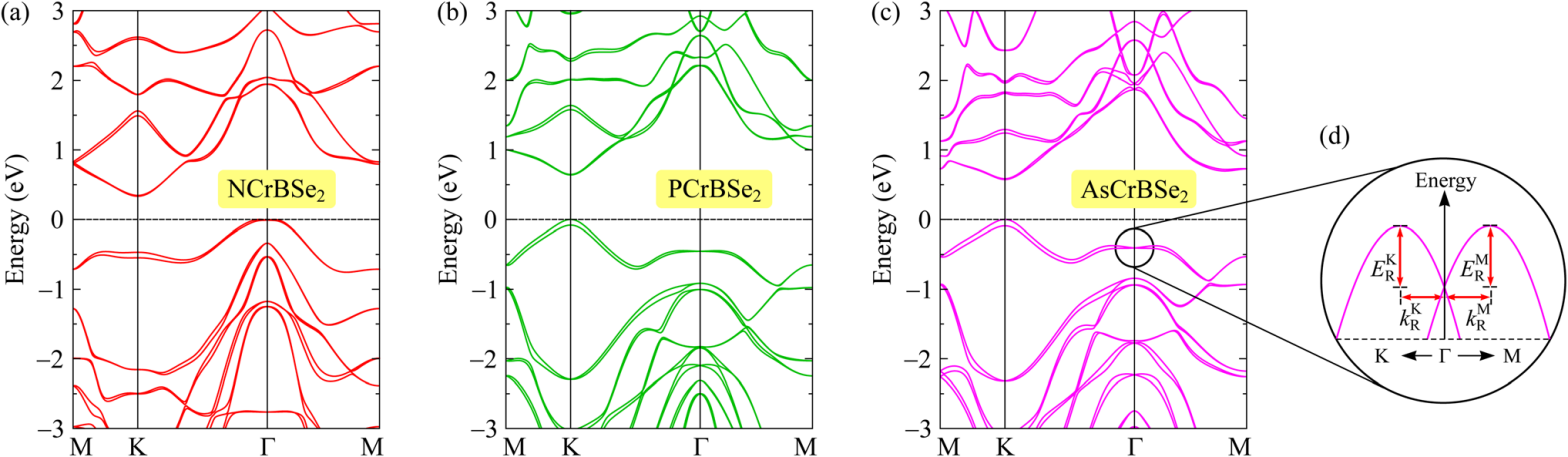
PBE+SOC band structures of NCrBSe_2_ (a), PCrBSe_2_ (b), and AsCrBSe_2_ (c) monolayers. (d) Illustration of Rashba-type spin splitting at the *Γ* point of the valence band.

SOC plays a fundamental role in determining the electronic properties of 2D materials by reshaping the band structure, inducing spin splitting, and governing spin- and valley-dependent phenomena. Its inclusion is particularly crucial when investigating Janus materials. Owing to their asymmetric atomic configuration, Janus structures intrinsically break out-of-plane mirror and inversion symmetries, generating an internal electric field. When combined with the presence of relatively heavy constituent elements, this symmetry breaking can markedly enhance SOC effects, resulting in pronounced spin splitting in the electronic bands. As a consequence, SOC can significantly affect key electronic characteristics, including band-gap magnitude, band dispersion, spin polarization, and the emergence of Rashba- or Dresselhaus-type effects. Neglecting SOC may therefore lead to an incomplete or inaccurate description of the electronic behavior of Janus materials, especially in the context of spintronic and valleytronic applications. In Fig. 4, we show the PBE+SOC band structures of the proposed 2D Janus ZCrBSe_2_ (Z = N, P, As) monolayers. It is indicated that when SOC is taken into account, spin-degenerate bands split at high-symmetry points of the Brillouin zone where inversion symmetry is broken. A pronounced Zeeman-type spin splitting is observed in the valence band at the *K* point of the Brillouin zone. The calculated results indicate that the spin splitting energy *λ*^v^_Z_(*K*) in the valence band at the *K* point amounts to 79.4, 81.2, and 90.9 meV for NCrBSe_2_, PCrBSe_2_, and AsCrBSe_2_ monolayers, respectively. This sizable splitting highlights the significant role of spin–orbit coupling and broken inversion symmetry in shaping the spin-resolved electronic structure of these materials. In addition, the inclusion of SOC leads to a slight reduction in the band-gap widths of the studied materials. The calculated results indicate that the PBE+SOC band gap energies of NCrBSe_2_, PCrBSe_2_, and AsCrBSe_2_ monolayers decrease to 0.75, 0.64, and 0.57 eV respectively, as summarized in Table 2. This reduction reflects the influence of SOC on the electronic states near the band edges and highlights the importance of SOC in accurately describing the electronic properties of these structures.

Furthermore, the SOC effect not only leads to a reduction of the band gap and induces Zeeman-type spin splitting in the monolayer, but also triggers a pronounced Rashba-type spin splitting in the valence band around the *Γ* point, as illustrated in Fig. 4. This Rashba splitting originates from the combined action of strong spin–orbit coupling and the broken inversion symmetry of the monolayer, resulting in momentum-dependent spin splitting with characteristic spin–momentum locking. From a quantitative perspective, the Rashba effect is characterized by three fundamental parameters, including the Rashba energy *E*_R_, the momentum offset *k*_R_, and the Rashba parameter *α*_R_. The Rashba energy measures the energy separation induced by spin–orbit interaction, while the momentum offset describes the shift of the spin-split band extrema in reciprocal space. The Rashba parameter, which combines both quantities, serves as a comprehensive indicator of the strength of Rashba spin splitting and provides direct insight into the efficiency of spin–momentum locking in the system. The Rashba parameter *α*_R_ can be evaluated through the expression^29,30^2
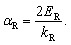


The calculated Rashba parameters of the Janus ZCrBSe2 monolayers are summarized in Table 2. Among the investigated systems, the AsCrBSe2 monolayer exhibits a relatively large Rashba splitting. In particular, the Rashba energies along the *ΓM* and *ΓK* directions are *E*^R^*M* = 32.3 meV and *E*^R^*K* = 30.2 meV, respectively, indicating a pronounced spin–orbit-induced band splitting near the band extrema. As shown in Table 2, no significant differences are observed in either the Rashba energy *E*_R_ or the momentum offset *k*_R_ between the *ΓM* and *ΓK* directions for all three Janus configurations. This near isotropy implies that the Rashba effect in these monolayers exhibits only a weak dependence on the crystallographic direction within the Brillouin zone, reflecting the underlying symmetry of the electronic structure. Furthermore, the Rashba parameters *α*_R_^*M*^ for the Janus NCrBSe_2_, PCrBSe_2_, and AsCrBSe2 monolayers are calculated to be 240.96, 97.30, and 290.99 meV Å, respectively. Correspondingly, the Rashba parameters along the *ΓK* direction, *α*_R_^*K*^, are 261.11, 102.33, and 296.08 meV Å for NCrBSe_2_, PCrBSe2, and AsCrBSe2, respectively. Notably, the *α*_R_^*K*^ values are slightly larger than their *α*_R_^*M*^ counterparts, suggesting a marginal enhancement of the Rashba coupling along the *ΓK* direction. The obtained Rashba parameters for the Janus *Z*CrBSe2 monolayers are comparable with those for Janus transition metal dichalcogenides, such as MoSSe (*α*_R_^*M*^ = 148 meV Å and *α*_R_^*K*^ = 158 meV Å), WSSe (*α*_R_^*M*^ = 157 meV Å and *α*_R_^*K*^ = 158 meV Å), or WSTe (*α*_R_^*M*^ = 324 meV Å and *α*_R_^*K*^ = 322 meV Å) monolayers.^29^ Overall, these results demonstrate strong and nearly isotropic Rashba spin splitting in Janus *Z*CrBSe_2_ monolayers, highlighting their potential for spintronic applications where robust and direction-insensitive spin–orbit coupling is desirable.

To fully understand the properties of materials, particularly those with intrinsic vertical asymmetry, it is essential to move beyond band structure analysis and conduct a rigorous examination of the electron work function. Integrating band characteristics with work-function behavior provides a more holistic view of the material's electronic structure and interfacial dynamics. Work function is a decisive factor in surface reactivity, interfacial energy-level alignment, and the efficiency of charge transport in optoelectronic applications. Due to their inherent lack of out-of-plane symmetry, Janus monolayers possess a natural, built-in dipole moment perpendicular to the atomic plane. This dipole creates an internal electrostatic field that induces an asymmetric potential distribution across the material. Consequently, the two distinct surfaces of the Janus slab exhibit different work functions. These surface-specific values are critical for determining interfacial charge transfer, electrode contact resistance, and band offset alignment within heterostructures. To quantify the work function *Φ*, we calculate the planar-averaged electrostatic potential along the *z*-direction (out-of-plane). The work function is defined as the energy required to move an electron from the Fermi level *E*_*F*_ to the vacuum level *E*_vac_:

3 *Φ* = *E*_vac_ − *E*_F_.

**Fig. 5:**
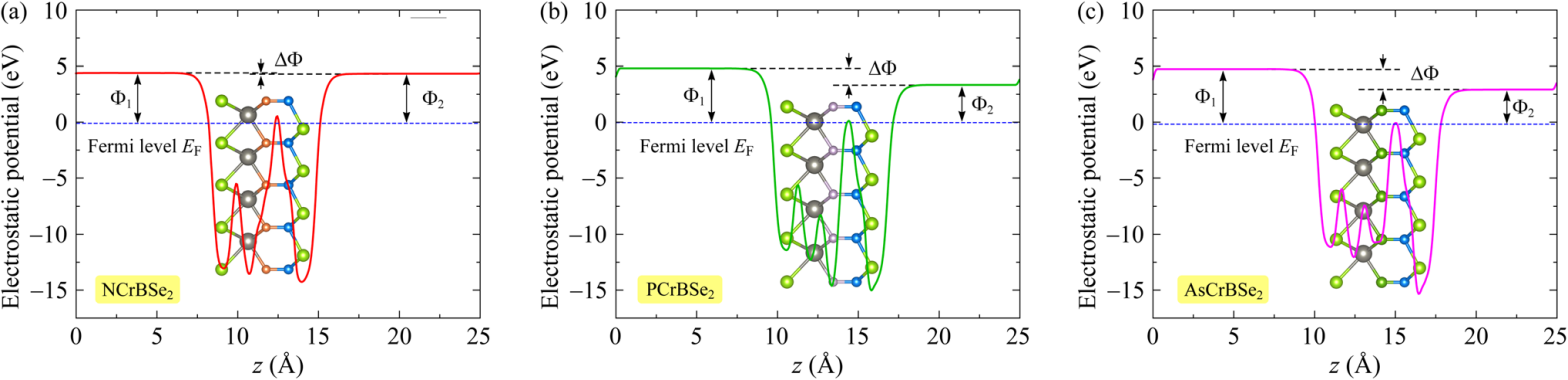
Electrostatic potential profiles of the 2D Janus NCrBSe_2_ (a), PCrBSe_2_ (b), and AsCrBSe_2_ (c) monolayers.

The planar-averaged electrostatic potential profiles for Janus *Z*CrBSe_2_ monolayers are presented in Fig. 5. A pronounced divergence in the vacuum energy levels between the two opposing surfaces is evident, directly manifesting the intrinsic out-of-plane asymmetry inherent to these Janus structures. As detailed in Table 2, the vacuum-level offset (Δ*Φ*) increases systematically across the series, rising from 0.07 eV for NCrBSe_2_ to 1.47 eV for PCrBSe_2_ and 1.84 eV for NCrBSe_2_. This trend suggests that the internal dipole strength and the resulting electrostatic field are progressively enhanced as the atomic number of the Z element increases. Furthermore, the work function of the bottom surface (*Φ*_1_) is consistently higher than that of the top surface (*Φ*_2_), confirming a robust vertical compositional gradient. This asymmetric potential landscape is highly advantageous for tuning band alignments and enabling directional carrier transport. Specifically, the lower work function on the top surface implies that electron emission and interfacial charge transfer are energetically favored on that side, suggesting high potential for applications in photocatalysis and asymmetric contact engineering.

### 3.3 Carrier mobility

In this final section, we present a comprehensive analysis of carrier mobility of the proposed Janus ZCrBSe_2_ monolayers, derived by explicitly accounting for the distinct contributions of various scattering mechanisms. To ensure high predictive accuracy, our calculations were performed using the AMSET package.^16^ This computational framework interfaces directly with the VASP software, allowing for a first-principles evaluation of transport properties without relying on empirical parameters. The AMSET approach captures the complex interplay between charge carriers and the crystal lattice by modeling multiple scattering processes, including acoustic deformation potential (ADP) scattering, impurity (IMP) scattering, piezoelectric (PIE) scattering, and polar optical phonon (POP) scattering. The total carrier mobility was determined by combining the individual scattering contributions according to Matthiessen's rule, which assumes that different scattering mechanisms act independently and that their corresponding scattering rates are additive. The total mobility *µ*_total_ is mathematically defined by the summation of the reciprocal mobilities associated with each specific process:^10^4
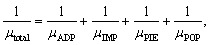
where *µ*_ADP_, *µ*_IMP_, *µ*_PIE_, and *µ*_POP_ correspond to the individual mobility contributions arising from acoustic deformation potential, impurity, piezoelectric, and polar optical phonon scattering processes, respectively.

The transport behavior of the material is fundamentally shaped by the carrier concentration, which directly modulates the interplay between carrier–phonon and carrier–impurity scattering mechanisms. In the present work, we systematically investigate the carrier mobilities of Janus ZCrBSe_2_ monolayers at two representative carrier concentrations of 1 × 10^16^ cm^−3^ and 1 × 10^20^ cm^−3^. These specific values are chosen to characterize the low- and high-carrier concentration cases, respectively, enabling a comprehensive assessment of the carrier transport behavior and the underlying scattering mechanisms across distinct doping conditions. Adopting carrier densities recommended by Xiao *et al.* for similar low-dimensional systems facilitates a direct comparison with existing theoretical benchmarks, thereby ensuring both the physical relevance and the internal consistency of our mobility calculations.^31^

**Fig. 6:**
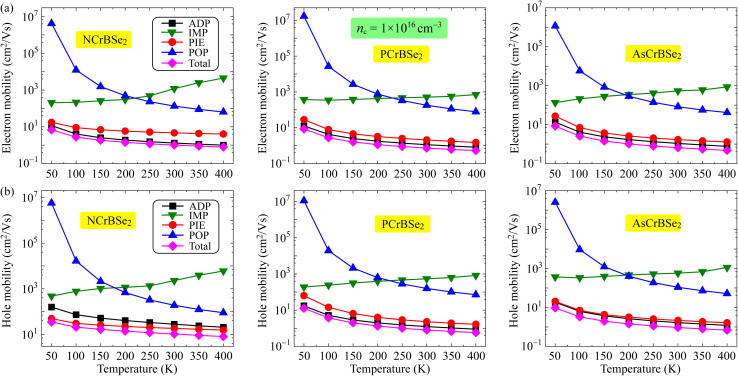
Temperature-dependent electron (a) and hole (b) mobilities in 2D Janus ZCrBSe_2_ monolayers at low carrier concentration of 1 × 10^16^ cm^−3^.

**Fig. 7:**
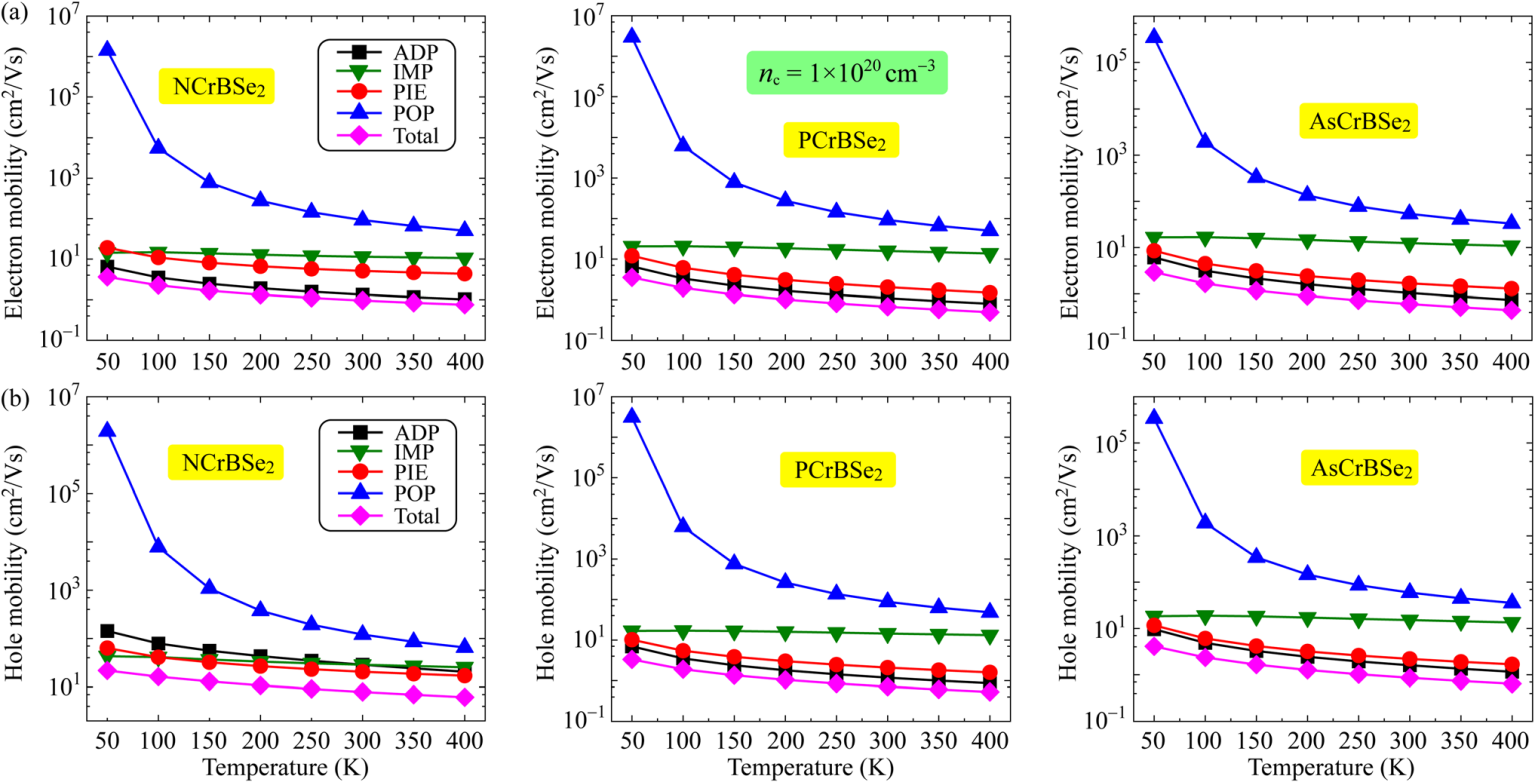
Temperature-dependent electron (a) and hole (b) mobilities in 2D Janus ZCrBSe_2_ monolayers at high carrier concentration of 1 × 10^20^ cm^−3^.

The temperature-dependent carrier mobility at a low concentration of 1 × 10^16^ cm^−3^ is illustrated in Fig. 6. Our calculations reveal that the electron mobility is primarily dictated by acoustic deformation potential (ADP) scattering. Furthermore, the contribution of piezoelectric (PIE) scattering to the total mobility is significant as displayed in Fig. 6(a). This trend remains consistent across all three materials. Meanwhile, a competition between IMP and ADP scattering processes is observed in determining the dominant role in hole mobility among the different structures. Specifically, while PIE scattering is the decisive factor in dictating the hole mobility of the NCrBSe_2_ monolayer, ADP scattering emerges as the key determinant for both PCrBSe_2_ and AsCrBSe_2_ monolayers as shown in Fig. 6(b). It is also noted that the distinct temperature dependencies observed for the various scattering mechanisms reflect the underlying physics of carrier transport in the Janus ZCrBSe_2_ monolayers at the low carrier concentration. While phonon-mediated processes such as ADP, PIE, and POP scattering become more intense at elevated temperatures due to the increased population of phonons, the IMP-limited mobility exhibits an inverse trend. The slight increase in IMP-limited mobility with temperature can be attributed to the increased thermal energy of the charge carriers. At higher temperatures, carriers possess higher average kinetic energy and velocity, allowing them to pass by ionized impurities more rapidly.^32^ Consequently, the scattering rate decreases, resulting in an enhancement of the IMP-limited mobility, a behavior that contrasts with the phonon-driven scattering mechanisms. It should be noted that the observed temperature dependence of *µ*_IMP_ is particularly characteristic of the low-carrier-density regime. However, this behavior evolves significantly as the system moves toward the high-concentration regime. At such high densities, the increased abundance of free carriers leads to strong electronic screening, which shields the long-range Coulomb potential of the impurities. Consequently, the relative dominance of IMP scattering diminishes, and the transport behavior becomes increasingly governed by the degenerate nature of the carrier gas and short-range scattering mechanisms. The temperature-dependent carrier mobility of ZCrBSe_2_ monolayers at the high carrier concentration of 1 × 10^20^ cm^−3^ is displayed in Fig. 7. The calculated results indicate that both the total carrier mobility and the mobilities associated with individual scattering mechanisms for charge carriers, including electrons and holes, decrease with increasing temperature. This temperature-dependent degradation of mobility arises from the enhanced carrier–phonon interactions at elevated temperatures. Similar to the behavior observed at low carrier concentrations, the electron mobility in the high carrier concentration regime is predominantly governed by ADP scattering, as illustrated in Fig. 7(a). In addition to ADP scattering, PIE scattering contributes significantly to determining the mobilities of both electrons and holes, as shown in Fig. 7(b), highlighting its importance in the overall carrier scattering landscape at high carrier concentrations.

To further evaluate the practical charge-transport performance of the material under realistic operating conditions, we subsequently examine the carrier mobility at room temperature. As room temperature represents the typical working environment for most electronic and optoelectronic devices, the carrier mobility at this temperature serves as a crucial figure of merit, directly influencing charge transport efficiency, switching speed, power consumption, and overall device performance. Table 3 summarizes the carrier mobilities arising from individual scattering mechanisms together with the total mobility at room temperature. Among all scattering channels, the mobility limited by acoustic deformation potential (ADP) scattering is the smallest, indicating that ADP scattering plays a dominant role in determining the total mobility, as dictated by Matthiessen's rule [Eq. (4)]. Consequently, the proposed Janus ZCrBSe_2_ monolayers exhibit relatively low total carrier mobilities at room temperature. In the low carrier concentration case of 1 × 10^16^ cm^−3^, the total electron mobilities of NCrBSe_2_, PCrBSe_2_, and AsCrBSe_2_ are 1.01, 0.73, and 0.65 cm^2^ V^−1^ s^−1^, respectively, and show a slight reduction with increasing carrier concentration.

**Table 3:** Total carrier mobility and carrier mobilities limited by individual scattering mechanisms in ZCrBSe_2_ (Z = N, P, As) monolayers at room temperature. Carrier concentration *n*_c_ is given in cm^−3^ and all mobilities *µ* are reported in cm^2^ V^−1^ s^−1^

Carrier concentration	Material	Carrier type	*µ*_ADP_	*µ*_IMP_	*µ*_PIE_	*µ*_POP_	*µ*_total_
*n*_c_ = 1 × 10^16^	NCrBSe_2_	Electron	1.30	1.20 × 10^3^	4.67	134.00	1.01
	PCrBSe_2_		1.13	512.00	2.08	180.00	0.73
	AsCrBSe_2_		1.08	538.00	1.69	82.80	0.65
	NCrBSe_2_	Hole	27.90	2.28 × 10^3^	18.30	189.00	10.39
	PCrBSe_2_		1.24	540.00	2.31	158.00	0.80
	AsCrBSe_2_		1.66	578.00	2.15	109.00	0.93
*n*_c_ = 1 × 10^20^	NCrBSe_2_	Electron	1.33	11.40	5.10	92.00	0.96
	PCrBSe_2_		1.09	15.80	2.03	92.20	0.67
	AsCrBSe_2_		1.05	12.50	1.69	53.70	0.61
	NCrBSe_2_	Hole	28.70	28.70	20.50	121.00	7.89
	PCrBSe_2_		1.18	14.50	2.09	87.70	0.71
	AsCrBSe_2_		1.60	15.20	2.20	59.60	0.86

## 4 Conclusion

In conclusion, we have introduced a novel family of Janus ZCrBSe_2_ (Z = N, P, As) materials and systematically explored their physical properties *via* first-principles calculations. Our findings confirm that all investigated monolayers are semiconductors characterized by both dynamical and mechanical stability. A significant Rashba-type spin splitting occurs at the *Γ* point within the valence band, driven by the intrinsic out-of-plane inversion asymmetry. Notably, the Janus AsCrBSe_2_ monolayer exhibits a substantial Rashba parameter of approximately 290 meV Å, highlighting its promise for spintronic devices. Furthermore, a rigorous assessment of carrier mobility, incorporating diverse phonon scattering mechanisms, identifies the dominant factors influencing charge transport. Our analysis indicates relatively low total mobilities for both electrons and holes, emphasizing the limiting effect of phonon scattering in these Janus structures.

## Conflicts of interest

There are no conflicts of interest to declare.

## Data Availability

All data that support the findings of this study are included within the article.
